# Trait-based mapping to identify the genetic factors underlying anaerobic germination of rice: Phenotyping, GXE, and QTL mapping

**DOI:** 10.1186/s12863-020-0808-y

**Published:** 2020-01-17

**Authors:** Sharmistha Ghosal, Fergie Ann Quilloy, Carlos Casal, Endang M. Septiningsih, Merlyn S. Mendioro, Shalabh Dixit

**Affiliations:** 1 International Rice Research Institute, Los Baños, Laguna Philippines; 2 University of the Philippines, Los Baños, Laguna Philippines; 3 Bangladesh Rice Research Institute, Gazipur, Bangladesh; 4 Soil & Crop Science, Texas A&M University, 2474 TAMU, College Station, TX USA

**Keywords:** Rice, Anaerobic germination, BSA, QTL, DSR

## Abstract

**Background:**

Anaerobic germination is one of the most important traits for rice under direct-seeded conditions. The trait reduces risk of crop failure due to waterlogged conditions after seeding and allows water to be used as a means of weed control. The identification of QTLs and causal genes for anaerobic germination will facilitate breeding for improved direct-seeded rice varieties. In this study, we explored a BC_1_F_2:3_ population developed from a cross between BJ1, an *indica* landrace, and NSIC Rc222, a high-yielding recurrent parent. The population was phenotyped under different screening methods (anaerobic screenhouse, anaerobic tray, and aerobic screenhouse) to establish the relationship among the methods and to identify the most suitable screening method, followed by bulk segregant analysis (BSA) to identify large-effect QTLs.

**Results:**

The study showed high heritability for survival (SUR) under all three phenotyping conditions. Although high correlation was observed within screening environments between survival at 14 and 21 days after seeding, the correlation across environments was low. Germination under aerobic and anaerobic conditions showed very low correlation, indicating the independence of their genetic control. The results were further confirmed through AMMI analysis. Four significant markers with an effect on anaerobic germination were identified through BSA. CIM analysis revealed *qAG1–2, qAG6–2, qAG7–4, and qAG10–1* having significant effects on the trait. *qAG6–2 and qAG10–1* were consistent across screening conditions and seedling age while *qAG1–2 and qAG7–4* were specific to screening methods. All QTLs showed an effect when survival across all screening methods was analyzed. Together, the QTLs explained 39 to 55% of the phenotypic variation for survival under anaerobic conditions. No QTL effects were observed under aerobic conditions.

**Conclusions:**

The study helped us understand the effect of phenotyping method on anaerobic germination, which will lead to better phenotyping for this trait in future studies. The QTLs identified through this study will allow the improvement of breeding lines for the trait through marker-assisted selection or through forward breeding approaches such as genomic selection. The high frequency of the BJ1 allele of these QTLs will enhance the robustness of germination under anaerobic conditions in inbred and hybrid rice varieties.

## Background

Direct seeded is increasingly becoming an important cultivation method across rice growing areas. Particularly in Asia, large areas traditionally grown under puddled transplanted system are shifting to direct seeded systems. This is mainly due to the shortage of water and labor in these areas. While, the cultivation shifts, the varieties which are developed for transplanted systems are used under direct seeded conditions and become susceptible to challenges specific to the system. One of the major traits required in rice varieties to be successful under direct seeded systems is the ability to produce good crop stand despite changing seasonal conditions at early stages. In the absence of such an ability, farmers are forced to use high seed rates as a risk management strategy in case of poor germination. This is not only costly in case inbred seeds are purchased but may also leads to higher disease and pest pressure due to dense and uneven planting. Further, such a risk practically makes the use of hybrid technology impossible due to high costs associated to the seeds. Of the several germination traits needed for direct seeded rice, anaerobic germination (AG) is the most important. AG refers to the ability of plants to germinate and develop roots and shoots under water. In rice, this becomes exceedingly important in direct seeded environments where flooding could occur immediately after seeding due to improper field leveling and/or heavy rain fall. While most rice genotypes fail to germinate under water, there exists considerable genetic variation among landraces for AG. In recent years, a series of linkage mapping studies identified many QTLs with major and minor effects on AG [1–6]. Among the identified AG QTLs, qAG9–2 on chromosome 9, has been fine-mapped to OsTPP7 which was found to be responsible for starch mobilization, embryo germination and coleoptile elongation [7]. While several studies have attempted to study the trait, much about the mechanism of AG remains unknown due to the complex nature of the trait. Germinaiton to anaerobic conditions is known to have numerous physiological processes involved these include seed longevity, seedling vigor [4, 8–12], seedling growth and adjustment of carbohydrate metabolism [13–15], fast coleoptile elongation, fast leaf and root development [7, 16] and high carbohydrate reserve of seed [17–19]. Further genetic studies are thus required to better understand the trait.

Modern-day plant breeding requires the identification, validation, and rapid integration of large-effect QTLs into breeding programs. While small-effect QTLs can be efficiently managed through population improvement methods such as genomic selection (GS), the rapid identification and deployment of large-effect QTLs may give breeding pipelines a jump-start. This requires the scanning of larger populations derived from different donors and recipients to be able to identify the most robust and consistent QTLs. Techniques such as bulk segregant analysis (BSA) are applicable in working with several mapping populations simultaneously and allow identification of QTLs with consistency across recipient backgrounds which is one of the major requirements for a QTL to be useful in breeding programs. Apart from this, the technique favors the detection of large-effect QTLs, thus maintaining the focus on robustness and consistency. While BSA has been used for QTL mapping for several traits, identification of genomic regions underlying AG has not been explored. Seedling-stage phenotyping allows the screening of large populations for AG at a time. If successful, this technique, combined with high-throughput phenotyping, may allow the simultaneous scanning of several biparental or multi-parental populations, thus leading to the rapid discovery of large-effect QTLs underlying the trait. In this study, we aimed to use a BC_1_F_2:3_ population, developed by crossing BJ1 with high AG potential as the donor parent with NSIC Rc222 an AG susceptible but high yielding line as the recipient parent, for BSA to identify QTLs related to AG. The study also aimed to understand the interactions between the identified QTLs and determine complementary QTL classes that could be useful for breeding activities. Moreover, we aimed to establish the relationship among the different screening conditions used in the study and determine the most stable breeding lines and their QTL combinations in methods for AG QTL detection.

## Results

### Phenotypic variation and correlation among traits

**Fig. 1:**
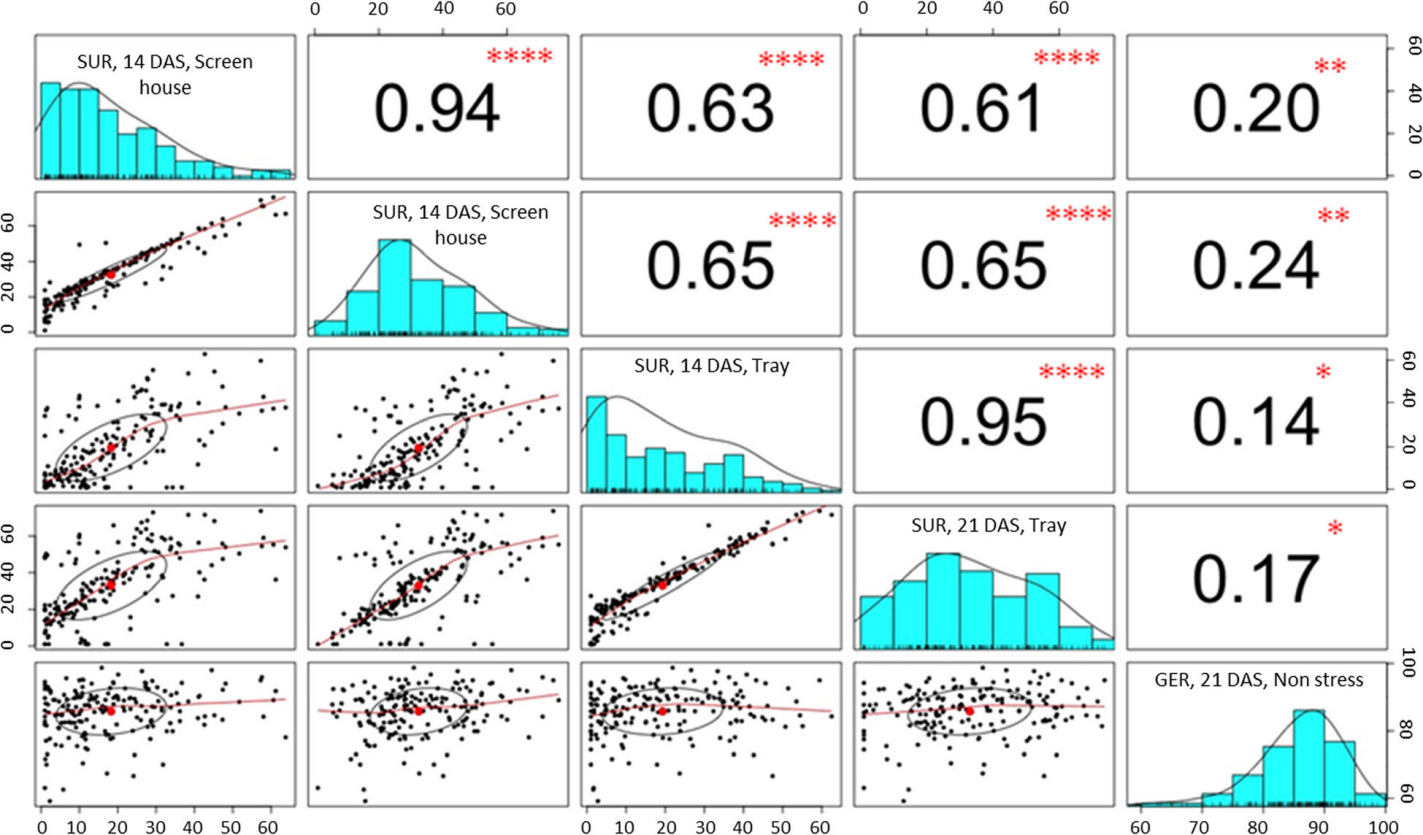
Phenotypic distribution and Pearson correlation coefficients between traits for survivability under screen house, tray and control conditions of the BC_1_F_2:3_ mapping population of BJ1/NSIC Rc222. *, **, **** = significant at 5, 1, 0.01% *P* levels, respectively

The population along with its parents was analyzed for phenotypic performance under various screening conditions, including anaerobic conditions in a screenhouse and in trays and aerobic conditions in a screenhouse. Table 1 presents the results of analysis of variance (ANOVA) for survivability under both anaerobic environments and germination under a non-stress aerobic environment. Significant differences were observed among the genotypes for all traits with broad-sense heritability (H^2^) ranging from 0.77 to 0.88. The average survival rates of the parents (BJ1 and NSIC Rc222) under anaerobic conditions during germination in the screenhouse were from 39.6 to 50.9% and from 11.1 to16.2%, respectively, in screenhouse conditions at 14 and 21 days after seeding (DAS), while the population mean was 17.3 and 31.4%, respectively. The survival rates of the parents in tray screening were slightly lower, with BJ1 and NSIC Rc222 showing 29.7 to 41.2% and 3.7 to 11.2% for 14 and 21 DAS, respectively. The population mean, however, was slightly higher, with 18.3 and 32.0% survival for 14 and 21 DAS, respectively (Table 1). Both parents showed similar germination under control conditions, with BJ1 and NSIC Rc222 having 92.9 and 91.0% germination, respectively. A continuous frequency distribution was observed for all traits, with survival recorded with 21 DAS showing a more normal distribution than with 14 DAS (Fig. 1). Highly significant positive correlations were observed for survivability at different seedling ages within environments. However, the correlation was relatively lower across environments. Relatively higher correlation (0.61 to 0.65) was observed for germination under anaerobic conditions across screenhouse and tray conditions compared with non-stress and anaerobic conditions (0.14 to 0.24) (Fig. 1). A positive correlation, however, was observed between survivability under AG and germination under non-stress conditions, which indicates the effect of viability on anaerobic germination. However, the low degree of correlation between these traits indicates the independent genetic control underlying the two traits. High heritability for survivability was also observed for the trait, suggesting importance of the population for QTL mapping.

**Table 1:** Analysis of variance for survivability (SUR) under anaerobic conditions and germination (GER) under non-stress condition for BJ1/2*NSIC Rc222 at 14 and 21 days after seeding (DAS) for single experiments

	SUR (Screenhouse)		SUR (Tray)		GER (Non-stress)
	14DAS	21DAS	14DAS	21DAS	21 DAS
Population mean	17.3	31.4	18.3	32.0	84.8
BJ1	39.6	50.9	29.7	41.2	92.9
NSIC Rc222	11.1	16.2	3.7	11.2	91.0
H^2^	0.87	0.85	0.84	0.88	0.77
SED	7.3	8.1	8.4	9.1	8.1
P	****	****	****	****	****

*H*^*2*^ heritability (broad sense), *SED* standard error of difference, **** = significant at 0.01% *P* levels

### GXE interactions and AMMI analysis

**Fig. 2:**
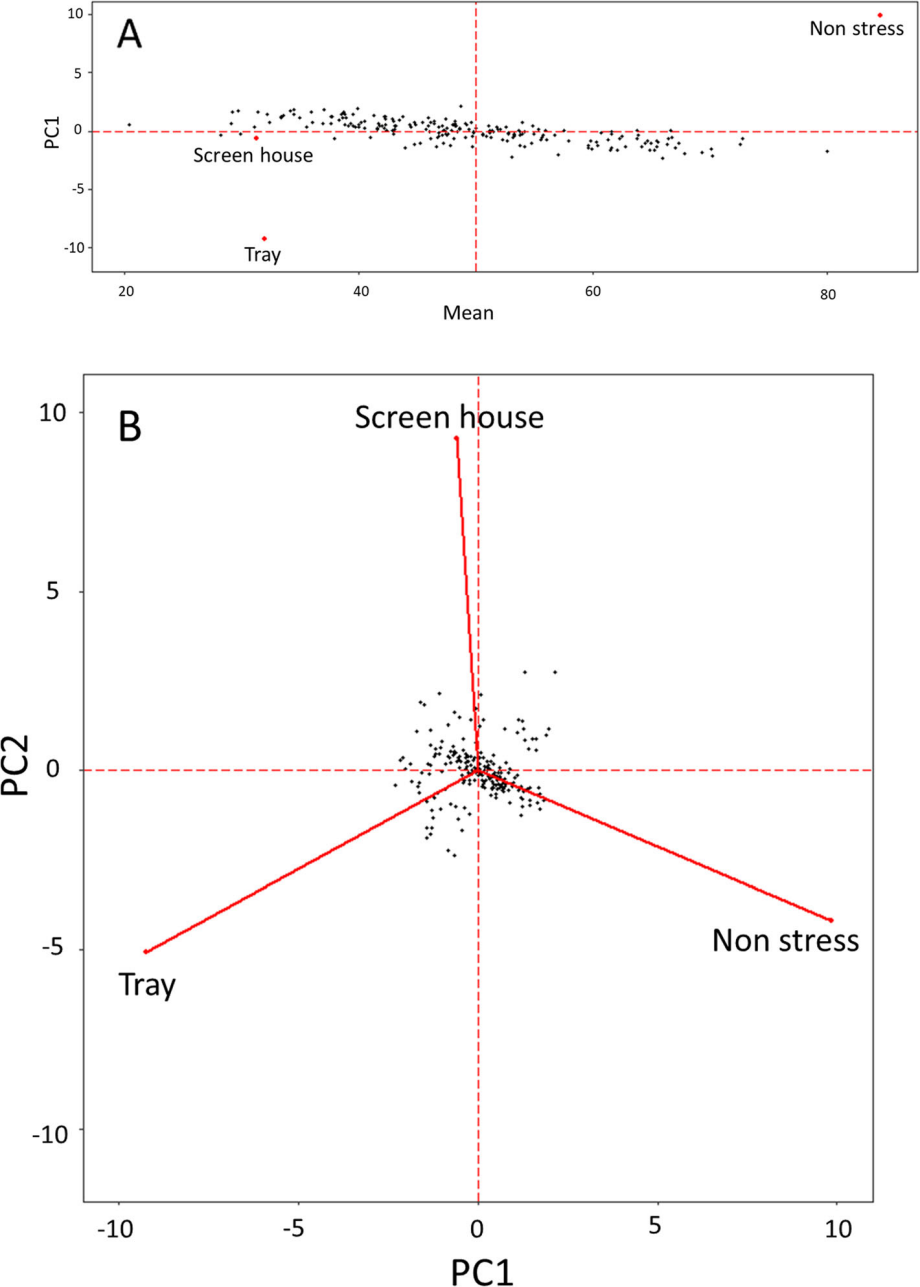
(A) AMMI-1 biplot of mean germination across 3 conditions and PC-1 scores (B) AMMI- 2 biplot of survivability (SUR) at 21DAS showing the stability of lines across screenhouse and tray screening for anaerobic germination and non-stress conditions in the screenhouse

Multi-environment analysis revealed a significant effect of the genotype and the genotype-by-environment (GXE) interactions for germination at both 14 and 21 DAS across environments (Table 2). Due to the significance of GxE interaction, further analysis with additive main effects and multiplicative interaction (AMMI) models was conducted to examine the relationship of the different genotypes and environments. AMMI showed that 66.6% of the sum of squares for interactions was explained by PC1 while the remaining 33.4% was explained by PC2 (Fig. 2). Figure 2a presents the AMMI-1 biplot for mean germination across the three conditions on the abscissa and PC1 scores of GXE interactions on the ordinate. Both anaerobic environments had similar means while the mean of the aerobic environment was much higher. The interaction patterns of all three environments were different from each other. Genotypes with PC score close to 0 are more stable across environments while those with higher fluctuation on either side of 0 have higher specificity for environments. Since stable germination across environments is required in this case, lines with a high mean across locations and PC value close to 0 are desirable. The analysis revealed similar differences among the three screening environments in terms of performance of the lines. However, the two anaerobic environments showed higher similarities to each other than that between anaerobic and aerobic screening conditions (Fig. 2b). The means derived from single trial analysis as well as those derived from the GXE and AMMI analyses were used for the QTL analysis.

**Table 2:** Analysis of variance for germination across screen house and tray anaerobic conditions and aerobic non-stress condition

Sources of variation	SUR (21DAS)			SUR (14DAS)		
	Df	SS	MS	Df	SS	MS
ENV	2	3,890,411	1,945,206	2	6,121,966	3,060,983
GEN	206	673,367	3269****	206	545,763	2649****
ENV:GEN	1816	1,104,174	608****	1816	878,617	484****
Residuals	1236	2472	2	1236	2472	2

*ENV* environment, *GEN* genotype, *ENV:GEN* environment-by-genotype interaction, *Df* degree of freedom, *SS* sum of squares, *MS* mean squares *F* F ratio, ****: Significant at 0.01% level of significance

### Bulk segregant analysis (BSA) and QTL mapping

**Fig. 3:**
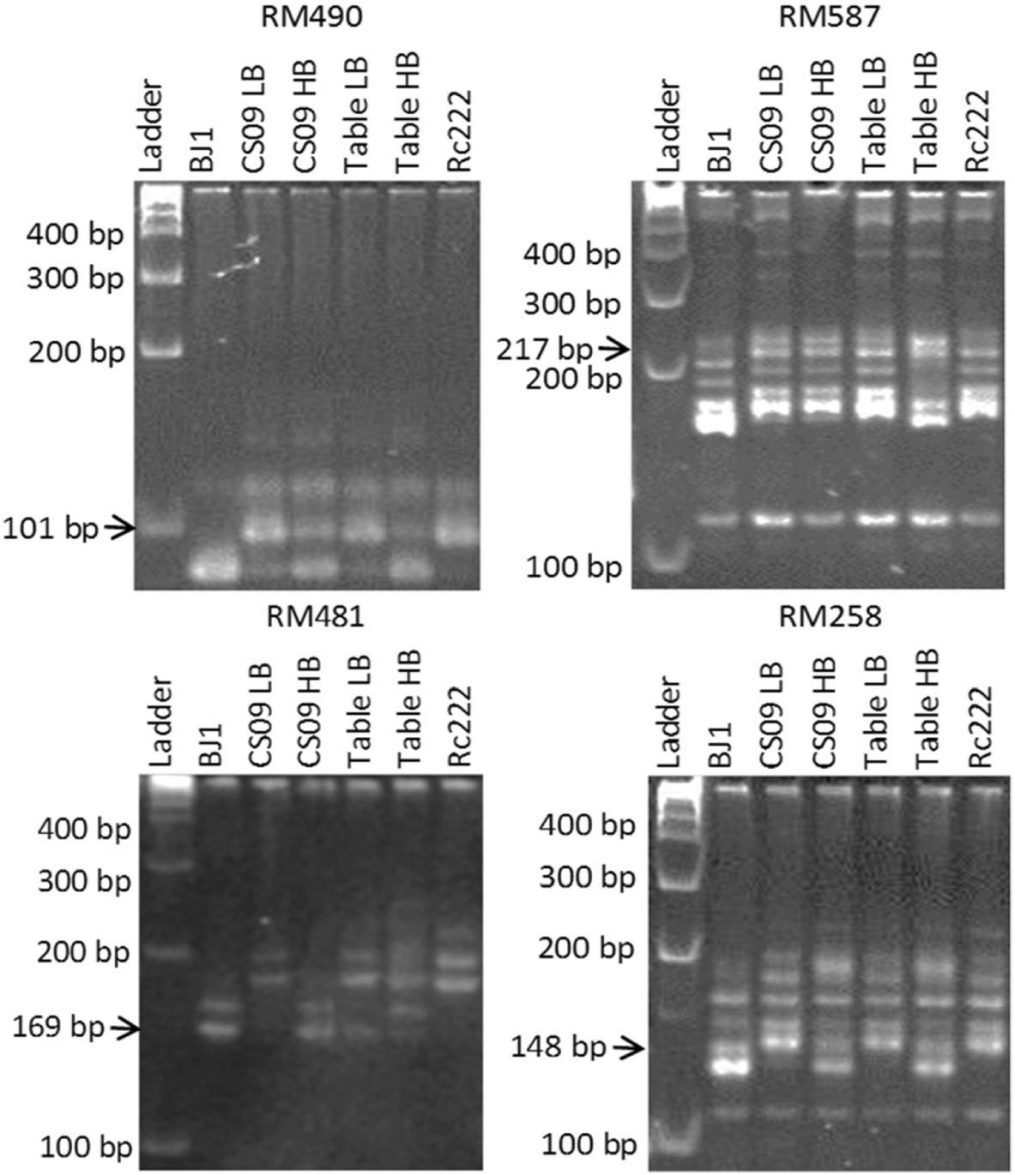
BSA results for the identified peak markers, viz. RM490 (*qAG1–2*), RM587 (*qAG6–2*), RM481 (*qAG7–4*), and RM258 (*qAG10–1*) for high and low bulks in two screening conditions along with tolerant (BJ1) and susceptible (NSIC Rc222) parents for survivability

A total of 102 clearly polymorphic markers were selected and run with four bulks (two each for survival under screenhouse and tray at 21 DAS) along with the two parents, among which a total of seven markers showing differences in banding patterns between bulks and the parents were further selected and used to genotype the whole population. Additional markers were added in each of the regions to facilitate composite interval mapping (CIM). Out of the seven markers, four showed clear polymorphism between bulks corresponding to the parent bands (Fig. 3). RM490 showed heterozygote bands for high bulks and NSIC Rc222 bands for low bulks. This was specifically clear for bulks developed for tray conditions. Similarly, RM587 showed clear polymorphism for the bulks developed for tray conditions. Contrary to this, RM481 showed a clearer polymorphism for bulks developed for screenhouse conditions. RM258 showed clear polymorphism for bulks developed for both conditions. Three other markers (RM148 on chromosome 3, RM296 on chromosome 9, and RM332 on chromosome 11) were also initially selected and used to genotype the full population. However, correspondence of the bulks to the parents was less clear for these markers than for the previous ones.

**Fig. 4:**
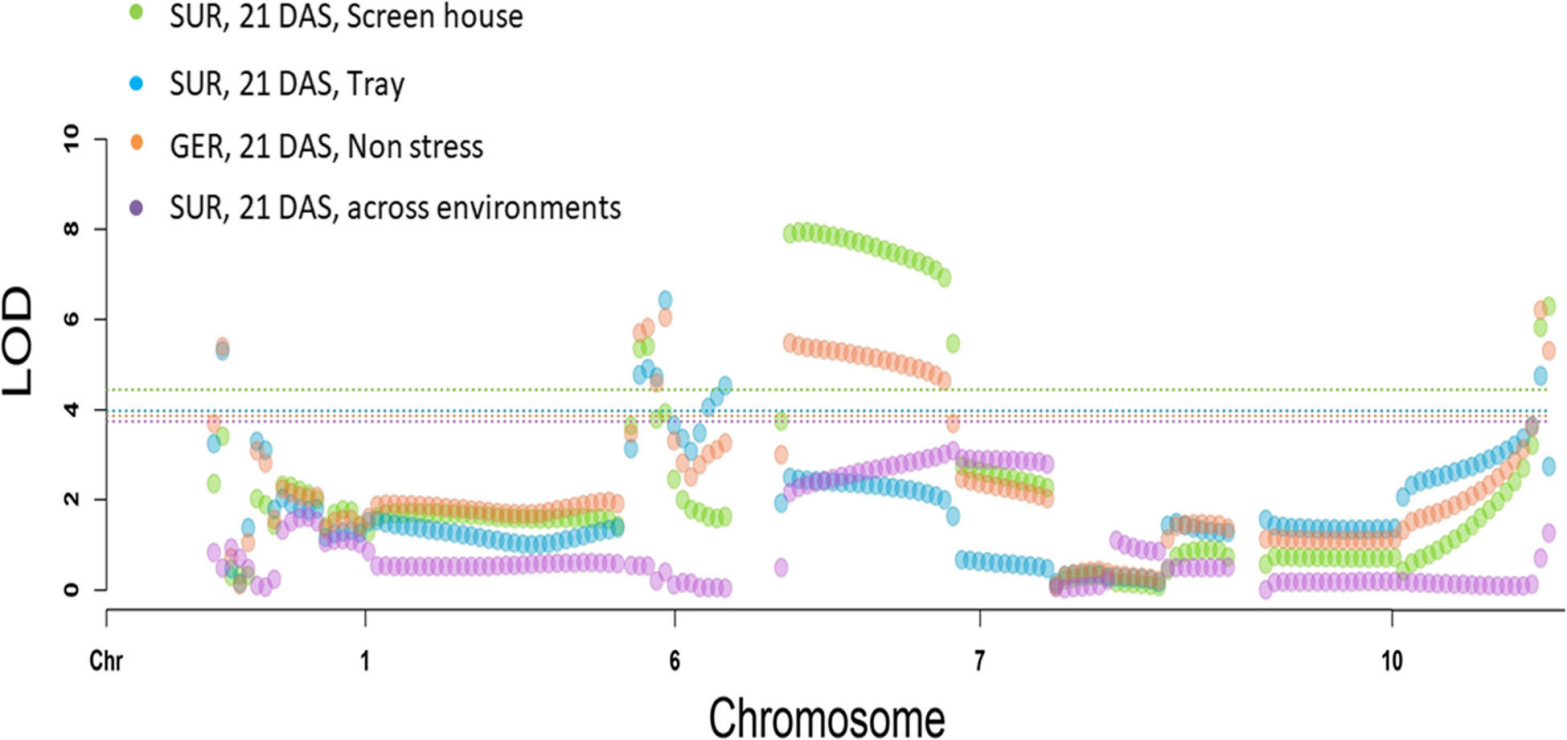
The QTL likelihood curves showing LOD values for *qAG1–2, qAG6–2, qAG7–4* and *qAG10–1* identified on chromosome 1, 6, 7 and 10 respectively through composite interval mapping using Q Gene 4.3.10

Results of CIM analysis are presented in Table 3. CIM showed the presence of four major QTLs controlling germination under anaerobic conditions in this population. All the QTLs were significant at the 1% level of significance. Under screenhouse conditions, three QTLs (*qAG6–2, qAG7–4*, and *qAG10–1*) explained a total of 43% of the phenotypic variation for the trait at 14 DAS and 46% of the phenotypic variation at 21 DAS (Table 3, Fig. 4). Under tray screening conditions, *qAG7–4* did not show any effect; however, *qAG6–2* and qAG10–1 showed significant effects on the trait. Another QTL (*qAG1–2*) on chromosome 1 was found significant for this condition. Altogether, the three QTLs explained 39% of the phenotypic variation for both 14 DAS and 21 DAS. None of the identified markers showed an effect under non-stress aerobic conditions. The genotype means across environments derived from the AMMI analysis was also used for conducting QTL analysis to identify the effect of the markers across environments. All four QTLs significantly affected germination across the different screening environments and explained 54% of the phenotypic variation at 14 DAS and 55% of the phenotypic variation at 21 DAS (Table 3, Fig. 4).

**Table 3:** List of QTLs detected for anaerobic germination potential in the BC_1_F_2:3_ mapping population of BJ1 and NSIC Rc222 significant at 1%level of significance

Trait	QTL	Chromosome	Locus name	Range	Position	A	LOD	R^2^
Screen house								
SUR (14DAS)	*qAG6–2*	6	RM190	RM508-RM510	5.5	9.0	5.6	0.13
	*qAG7–4*	7	RM481	RM481-RM21427	15.4	34.8	8.3	0.19
	*qAG10–1*	10	RM258	RM258-RM304	71.2	8.0	4.7	0.11
SUR (21DAS)	*qAG6–2*	6	RM190	RM508-RM510	5.5	8.9	5.4	0.13
	*qAG7–4*	7	RM481	RM481-RM21427	15.4	35.1	7.9	0.18
	*qAG10–1*	10	RM304	RM258-RM304	73.2	7.7	6.3	0.15
Tray								
SUR (14DAS)	*qAG1–2*	1	RM490	RM272-RM490	25.5	7.4	4.7	0.11
	*qAG6–2*	6	RM587	RM508-RM510	9.5	8.8	7.0	0.16
	*qAG10–1*	10	RM258	RM258-RM304	71.2	8.4	5.0	0.12
SUR (21DAS)	*qAG1–2*	1	RM490	RM272-RM490	25.5	9.0	5.3	0.13
	*qAG6–2*	6	RM587	RM508-RM510	9.5	10.3	6.4	0.15
	*qAG10–1*	10	RM258	RM258-RM304	71.2	9.9	4.7	0.11
Across environments								
SUR (14DAS)	*qAG1–2*	1	RM490	RM272-RM490	25.5	4.7	5.0	0.12
	*qAG6–2*	6	RM587	RM508-RM510	9.5	5.4	6.3	0.15
	*qAG7–4*	7	RM481	RM481-RM21427	13.4	10.3	6.0	0.14
	*qAG10–1*	10	RM258	RM258-RM304	71.2	5.9	5.6	0.13
SUR (21DAS)	*qAG1–2*	1	RM490	RM272-RM490	25.5	5.2	5.4	0.13
	*qAG6–2*	6	RM587	RM508-RM510	9.5	5.9	6.0	0.14
	*qAG7–4*	7	RM481	RM481-RM21427	13.4	10.5	5.5	0.13
	*qAG10–1*	10	RM258	RM258-RM304	71.2	6.9	6.2	0.15

*SUR* seedling survival (%), *DAS* days after seeding, *LOD* logarithm of odds, *R*^*2*^ phenotypic variation explained, *A* additive effects of the peak marker

### Line comparisons and QTL combinations

**Fig. 5:**
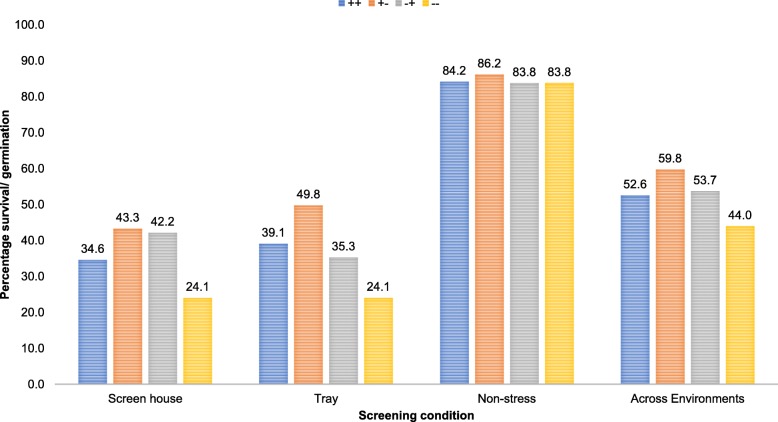
Effect of combination of allele types of qAG6–2 and qAG10–1 on survival under AG and germination under non-stress conditions. ++ (BJ1 allele at qAG6–2 and qAG10–1), + − (BJ1 allele at qAG6–2 and NSIC Rc222 allele at qAG10–1), − + (NSIC Rc222 allele at qAG6–2 and BJ1 allele at qAG10–1), −- (NSIC Rc222 allele at qAG6–2 and qAG10–1)

The population was further analyzed for the positive alleles at the peak markers of the four QTLs to understand the best QTL combinations. Figure 5 presents the mean survival under anaerobic and germination under AG and non-stress conditions along with that across screening environments for lines possessing various allele combinations of the two most consistent QTLs (qAG6–2 and qAG10–1) identified in this study. While QTL analysis showed the BJ1 allele to be the positive allele for all identified QTLs, the QTL combination analysis suggested otherwise for qAG6–2 and qAG10–1. In general, it was observed that survival under AG for lines with BJ1 allele for both QTLs was lower than those with one QTL with BJ1 allele. Lines with NSIC Rc 222 allele at both QTLs had the lowest survival under AG. No effect of QTL combinations was seen on germination under non-stress conditions. This was further confirmed with the analysis of allele patterns of the highest and lowest performing lines in the population (Table 4). The five most stable and high-performing lines and five of the most consistently low-performing lines across the three planting conditions for germination at 21 DAS are presented in Table 4. In all five best lines, at least one of the four QTLs was found to be fixed for the BJ1 allele (+). However, in three of the five cases, qAG6–2 contained NSIC Rc222 allele at qAG6–2 confirming the results of the QTL interaction analysis. *qAG1–2* and *qAG10–1* had the highest frequency of fixed BJ1 allele while *qAG7–4* was heterozygote in three out of the five best lines and fixed for the BJ1 allele for one line. Contrary to the most stable lines, those with low performance across environments showed the NSIC Rc222 allele (−) for most of the QTLs. The antagonistic nature of the two major QTLs may be a reason for better performance of the selected lines compared to BJ1 itself which possesses the tolerant alleles at both loci.

**Table 4:** Comparison of allele type for the four QTLs for lines with high and low germination across various AG screening methods

Lines	Screen house (AG)	Tray (AG)	Screen house (NS)	Across environments	PC1	PC2	qAG1–2	qAG6–2	qAG7–4	qAG10–1
IR 128754–161	73.4	72.7	94.0	80.1	−1.7	1.1	+	–	H	+
IR 128754–165	70.3	53.4	94.9	72.9	−0.7	1.6	H	H	H	+
IR 128754–81	75.1	54.3	88.1	72.4	−1.1	2.1	–	–	+	–
IR 128754–51	70.1	58.0	83.2	70.4	−1.5	1.8	+	–	H	–
IR 128754–178	57.2	70.6	83.3	70.2	−2.1	0.4	+	+	.	+
IR 128754–86	4.5	3.6	86.8	31.5	1.6	−0.9	–	–	–	–
IR 128754–101	4.8	0.0	84.4	29.7	1.7	−0.7	H	–	–	–
IR 128754–102	10.5	7.0	70.7	29.5	0.6	0.0	–	–	–	–
IR 128754–15	5.9	0.0	83.2	29.3	1.7	−0.5	–	H	–	–
IR 128754–136	0.0	0.0	62.2	20.4	0.6	−0.2	–	–	–	–
**NSICRc222**	**16.2**	**11.2**	**91.0**	**39.4**	**1.5**	**−0.5**	**–**	**–**	**–**	**–**
**BJ1**	**50.9**	**41.2**	**92.9**	**61.6**	**−0.1**	**0.8**	**+**	**+**	**+**	**+**

*AG* anaerobic germination, *NS* non-stress aerobic condition, *PC* principal component + BJ1 allele at peak marker, − NSIC Rc222 allele at peak marker, H heterozygous at peak marker, in *bold text* are the parents of the population

## Discussion

Trait-based mapping has been used in the past to identify QTLs for tolerance of a variety of abiotic stresses. In particular, BSA has been used in rice for the identification of QTLs for stresses such as heat, drought, cold, and salinity [20–24]. The technique, however, has never been used for traits such as anaerobic germination and early vigor. This study presents the use of trait-based mapping for the identification of stable QTLs for anaerobic germination in rice. We conducted an extensive phenotyping of a BC_1_F_2:3_ mapping population under two different setups (screenhouse and tray) imposing anaerobic conditions during germination and one germination testing under normal conditions. Results from the phenotypic analysis showed significant differences among the lines under all three phenotyping setups and high heritability for all the measured traits (Table 1). More normal distribution was observed for germination measured at 21 DAS than at 14 DAS, which indicates that the 21 DAS parameter is more suitable and accurate (Fig. 1).

High correlation for germination was observed within the screening environments for the two dates. However, the correlation was low across the tray and screenhouse conditions. Further, low correlation was observed between germination under normal and anaerobic conditions, indicating the independence of genetic control between the two traits (Fig. 1). Screenhouse phenotyping, which imposes conditions closer to the field setup, was found more suitable for screening for anaerobic germination than the tray setup. The AMMI analysis confirmed this finding, for which all three screening conditions were found different from each other (Fig. 2). In such a scenario, the screening method closest to natural field conditions is the most reliable. The study thus shows the suitability of the screenhouse screening method as the best for rapid screening of a large number of lines in a small area.

We used BSA as the method of genotyping for this study. BSA was initially designed to target major-effect QTLs; however, its continued advances in combination with high-throughput genotyping technologies have increased its resolution to detect many underlying genetic factors, including minor causal alleles [25]. In this study, BSA revealed four markers on chromosomes 1, 6, 7, and 10 where the DNA bulks had clear correspondence with the parents (Fig. 3). All four markers detected the presence of QTLs through CIM analysis. Three other markers were also identified in the first round of BSA but these markers did not show bulk-to-parent correspondence as with the first four. Moreover, no significant QTLs were identified at these loci upon QTL mapping.

BSA proved to be a convenient method to identify major QTLs that explain a large proportion of the phenotypic variation for traits, with minimum genotyping. The use of SSR markers, however, limited the information on the QTL region due to a low number of polymorphic markers in the marker regions. The identified QTLs will need further fine mapping to reduce the span of the QTL regions and undertake further studies. Flexible SNP genotyping platforms that can genotype a population using selected SNPs may provide much more precise results with smaller QTL spans. In particular, for traits related to germination, where phenotyping is relatively fast, rapid progress can be made by combining precise phenotyping methods with flexible SNP platforms to identify large-effect QTLs.

In our study, a total of four QTLs were identified (Table 3, Fig. 4) for AG. *qAG7–4* was the QTL with the largest effect but it was identified under screenhouse conditions only, for both 14 and 21 DAS. *qAG6–2 and qAG10–1* were identified across screenhouse and tray conditions at 14 and 21 DAS while *qAG1–2* was identified under tray conditions only. All four QTLs showed effects on mean germination across environments while there was none under aerobic non-stress conditions. The identified QTLs explained a range of 39 to 55% of the total phenotypic variation for the trait (Table 3). The specificity of some QTLs to particular screening conditions and the effect of others across these screening methods explain the genetics underlying the trait. Multiple component traits such as coleoptile length, higher starch reserves, and greater water imbibition are responsible for robust anaerobic germination across environments. The varying response of each of the QTLs toward screening conditions shows the requirement of a combination of such traits to achieve higher and more robust germination. It is likely that different component traits and physiological factors are controlled by genes underlying these QTLs, which are expressed in different screening conditions. Further, none of these QTLs showed their effect under non-stress conditions. This shows their specificity to anaerobic germination-related traits and the importance of these regions in breeding programs. However, this also means that these QTLs do not affect rice’s seedling germination per se. These QTLs should therefore be combined with other factors such as early vigor and seed viability to be able to develop lines with high robustness of germination across varying soil types and direct-seeding methods.

We conducted a QTL interaction analysis with qAG6–2 and qAG10–1 which showed a disadvantage if BJ1 allele for both QTLs were present together (Fig. 5). While, BJ1 allele was positive allele for all four QTLs in the CIM. Combining it for qAG6–2 and qAG10–1 led to lower germination compared to lines where either of the two QTLs had a NSIC Rc 222 allele. This was further confirmed by the comparison of QTL alleles in the five lines with the highest tolerance of anaerobic conditions during germination and the highest stability of germination across environments with those with the lowest tolerance and stability (Table 4). We could establish clearly that the presence of even one of the four QTLs was advantageous over the lines without any of the QTLs. Further, the combination of BJ1 allele at qAG1–2 and qAG10–1 seemed to be the most advantageous while similar to the QTL class analysis, presence of NSIC Rc 222 allele at qAG6–2 was more advantageous. The two analysis together indicate that the pyramiding of BJ1 allele at qAG1–2 and qAG10–1 with NSIC Rc 222 allele at qAG6–2 may lead to highest advantage. The development of lines with varying combinations of these QTLs will allow us to understand their interaction patterns in more detail.

## Conclusions

The present study reports the phenotyping and QTL mapping of a BC_1_F_2:3_ mapping population evaluated in two different screening conditions, which identified a total of four QTLs. Our study showed the effect of phenotyping methods for anaerobic germination on the performance of the lines. Statistical analysis revealed lower correlation of survival across screening methods than for lines within the same screening methods at different times. A total of four QTLs were identified in this study on chromosomes 1, 6, 7, and 10. The QTLs on chromosomes 6 and 10 showed effects across screening conditions while those on chromosomes 1 and 7 were specific to screening methods. None of the QTLs showed effect on germination under non-stress conditions. The study allowed us to use robust phenotyping techniques to understand the anaerobic germination trait better and identify large-effect QTLs with stable effects on the trait. Increasing the frequency of the tolerant allele of these QTLs in breeding programs will allow the development of breeding lines with more stable germination across different conditions.

## Methods

### Mapping population

A BC_1_F_2:3_ population was generated by crossing BJ1, a parent from India with high anaerobic germination potential, and NSIC Rc222, a high-yielding but susceptible parent developed by IRRI. Both BJ1 and NSIC Rc222 belong to subspecies *indica*. These are medium-duration genotypes having growth duration of 110 days and 106 days, respectively. A total of 205 lines underwent phenotypic evaluation under flooding conditions during germination with the parents used as checks.

### Phenotyping and data collection

**Fig. 6:**
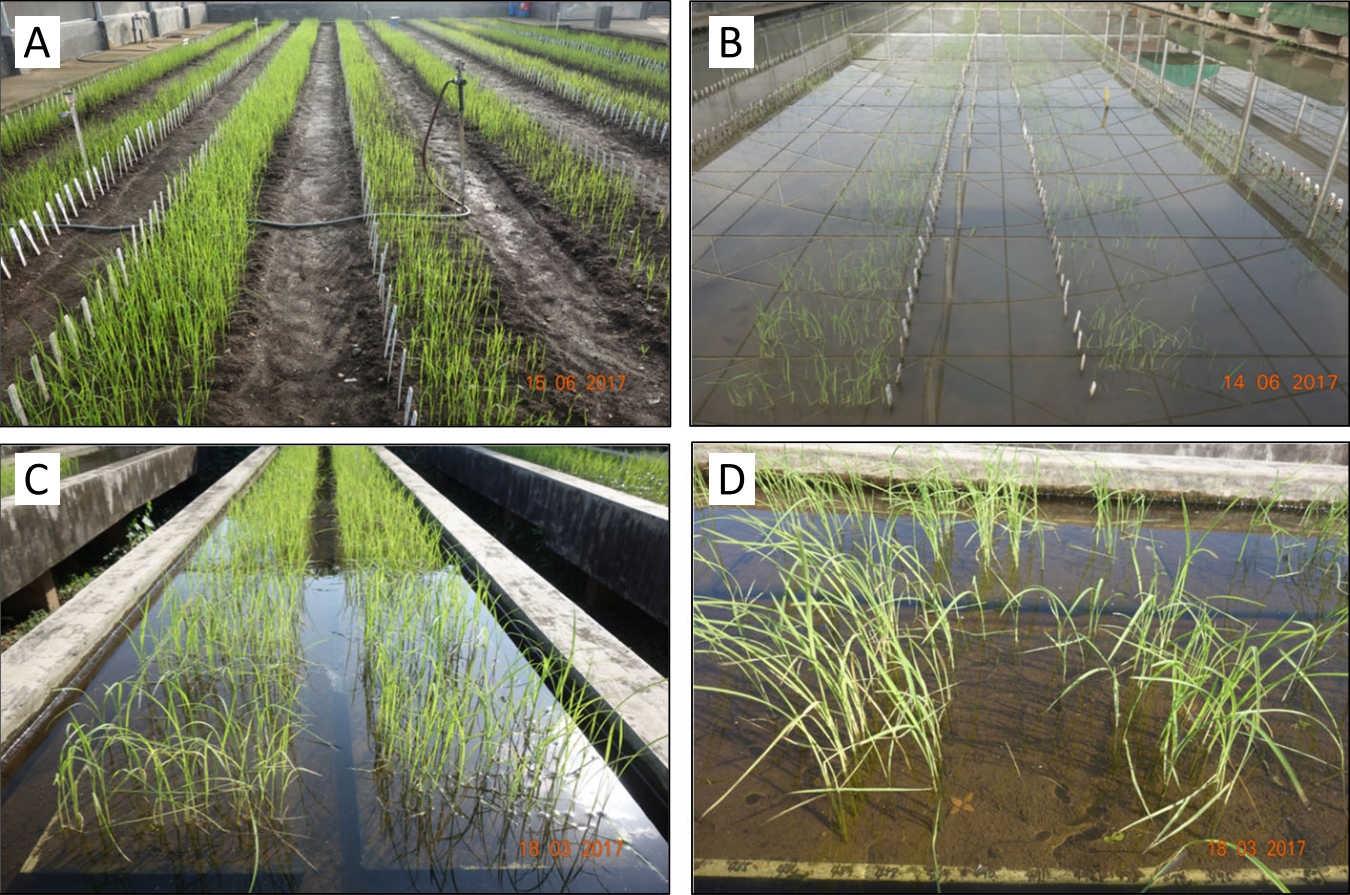
Phenotyping of the BC_1_F_2:3_ mapping population of BJ1 and NSIC Rc222. **a** Control experiment at 14 DAS in the screenhouse **b** stress experiment in the screen house at 14 DAS **c** stress experiment on the screening tray at 21DAS **d** variation for seedling survival under tray conditions

The population was screened under two conditions, one using trays filled with garden soil placed on the screening table and the other directly on the puddled soil of an IRRI screenhouse providing more natural field conditions for the phenotypic evaluation. A controlled experiment was also conducted using dry direct-seeded conditions in the screenhouse (Fig. 6). Freshly harvested dry seeds were placed in a hot-air oven set at 50 °C for 72 h to break the seed dormancy. An α-lattice design with three replications was followed in all cases.

For the tray screening, seedling trays (53.3 × 38.19 × 10.2 cm^3^) filled with fine garden soil were marked with a grid marker maintaining seeding depth of 1.0 cm with 15 lines per tray. Thirty seeds from each entry were sown in each line. After seeding, the lines were covered with garden soil. The trays were carefully submerged in concrete tables filled with 7–8 cm of water from the soil surface of the trays. This water depth was maintained for 21 days. Two measuring scales were placed in each table to constantly monitor and maintain the desired water level.

For the screenhouse screening, standard irrigated land preparation for wet field conditions was followed. After puddling and leveling, the excess surface water was drained and the area was left for 24 h to settle the soil. The field was then divided into beds in which 45-cm-long rows were laid out. These rows were drawn 10 cm apart with a depth of 1 cm using a grid marker. Thirty seeds from each entry were sown in each row and were covered with 1 cm of topsoil. The field was then slowly submerged in 7–8 cm of water above the soil surface. The water level was maintained as such for 21 days. Six measuring scales were placed at each corner and at the center of the field to monitor and maintain the desired water depth. Water temperature was monitored twice a day, once at 0700 and again at 1400.

For the controlled non-stress experiment in the screenhouse, standard land preparation for aerobic dry-soil conditions was followed. The protocol for layout and seed sowing was the same as it was in puddled soil except that the seeds were sown directly on the dry soil. After seeding, irrigation was done by overhead sprinklers. Three tensiometers were placed at equal distances inside the field to monitor soil moisture and thereby apply irrigation to maintain saturated soil conditions for 21 days.

The data on the number of surviving seedlings were recorded from both screening experiments by counting the number of seedlings that emerged above the water surface at 14 and 21 DAS.

### Statistical analysis

Survival rate (SUR) was calculated as the percent of the number of seedlings that survived relative to the total number of seeds used. ANOVA was conducted using PBTools V 1.4.0 [26]. The mixed linear model described below was used for α-lattice design analysis:

$$ {\mathrm{P}}_{ijk}=\upmu +{\mathrm{R}}_i+{B}_J\left({\mathrm{R}}_i\right)+{G}_k+{\mathrm{E}}_{ijk} $$

where P_ijk_ is the measurement recorded on a line, μ is the overall mean, R_i_ refers to the effect of the i^th^ replicate, B_j_ refers to the effect of the j^th^ block within the i^th^ replicate, G_k_ refers to the effect of the k^th^ genotype, and E_ijk_ refers to error effect. For the computation of means and standard error of difference (SED), the effects of replications and blocks within replications were considered as random, whereas, for the computation of variance components, the effects of genotypes, blocks, and replications were considered as random. Broad-sense heritability was calculated as

$$ {H}^2=\frac{\sigma_G^2}{\left({\sigma}_G^2+{\sigma}_E^2\right)/R} $$

where *H*^2^ stands for broad-sense heritability, ${\sigma}_G^2$ for genetic variance, ${\sigma}_E^2$ for error variance, and R for the number of replications in the experiment.

Correlation among traits, the frequency distribution, and graphical visualization were done using RStudio with the packages “corrplot” [27] and “psych” [28].

GXE analysis was conducted using PBTools V 1.4.0 [26] using the model

$$ {y}_{ij kl}=\mu +{l}_j+{r}_{kj}+{b}_{lkj}+{g}_i+{(gl)}_{ij}+{e}_{ij kl} $$

where μ is the overall mean, l_j_is the effect of the j^th^ environment, r_kj_ is the effect of the k^th^ replicate within the j^th^ environment, b_lkj_ is the effect of the l^th^ block within the k^th^ replicate of the j^th^ environment, g_i_ is the effect of the i^th^ genotype, (gl)_ij_ is the effect of the interaction between the i^th^ genotype and the j^th^ environment, and e_ijkl_ is the error. The effects of genotype and interaction between genotype and environment were considered fixed while the other effects were considered random.

Stability of the genotypes across different environments was determined through the AMMI model [29, 30], which can be written as

$$ {y}_{ij}=\mu +{g}_i+{e}_j+\sum \limits_{k=1}^m{l}_k{u}_{ki}^{\ast }{v}_{kj}^{\ast }+{\varepsilon}_{ij} $$

where y_ij_ is the mean yield of the i^th^ genotype in the j^th^ environment, μ is the general mean yield, g_i_ is the i^th^ genotypic effect, e_j_ is the j^th^ location effect, l_k_ is the eigen value of the PCA axis, k. ${u}_{ki}^{\ast }$ and ${v}_{kj}^{\ast }$ are the i^th^ genotype and j^th^ environment PCA scores for PCA axis k, ε_ij_ is the residual error, and m is the number of PCA axes retained in the model. GXE analysis was conducted using the software PB tools while AMMI analysis was conducted using the R package “agricolae 1.2–8” [31].

### Genotyping, bulk segregant analysis, and QTL mapping

A total of 181 lines were used for BSA and subsequent genotyping to identify the QTLs. Leaf samples were collected from a bulk of 20 plants per family and lyophilized for DNA extraction. DNA was extracted by the modified miniprep CTAB (cetyl tri-methyl ammonium bromide) method [32]. The extracted DNA was quantified using a nano-drop spectrophotometer (Thermo Scientific, Wilmington, DE, USA) to a concentration of 25 ng/μL and the quality was checked using 1.2% agarose gel. Separate DNA bulks were developed based on the phenotypic performance of the populations under both screening conditions. For each screening condition, two bulks were prepared by pooling DNA of 4% of the lines with lowest survival at 21 DAS, low bulk (BL), and the second bulk was created using the DNA of 4% of the lines with highest survival at 21 DAS, high bulk (BH). These bulks were prepared by pooling equal amounts of DNA with similar concentration (25 ng/μL) from each of the lines within each group of low and high bulks. A parental polymorphism survey between NSIC Rc222 and BJ1 was carried out with 600 SSR markers [33–35]. Clearly polymorphic markers were selected for BSA.

PCR amplification was performed on 96-well plates in 10 μL total volume containing 25 ng/μL DNA template, 10x PCR buffer (containing 10 mM Tris–HCl, pH 8.3, 50 mM KCl, 3 mM MgCl_2_), 200 μM dNTPs, 5 μM of each forward and reverse primer, and 1 unit of *Taq* polymerase on a thermal cycler (G-Storm, United Kingdom, and Kyratec, Australia). The PCR profile used for amplification includes 3 min of initial denaturation at 94 °C followed by 35 cycles of denaturation at 94 °C for 45 s, annealing at 55 °C for 45 s and extension at 72 °C for 45 s, and a final extension at 72 °C for 10 min. Bromophenol blue loading dye (2 μL) was added to the PCR products, which were run in 8% polyacrylamide gels (C.B.C. Scientific, USA) along with a 1 kb^+^ ladder (Invitrogen, Catalog no. 10787026) for 2 h. Staining with Sybr® safe (Invitrogen, Catalog no. S33102) was done and the bands were visualized using Alpha Imager 1220 (Alpha Innotech, CA, USA). Allele scoring was done using A (tolerant parent), B (susceptible parent), and H (heterozygous) calls. The markers with bulk bands corresponding clearly to the parents were considered to be significant. Single marker analysis was done for the significant markers to find candidate markers. More markers were selected on both sides of the candidate markers and the full population was then genotyped with these markers for the identification of QTLs using composite interval mapping. CIM was performed using the software Q Gene 4.3.10 [36]. The LOD thresholds obtained correspond to an experiment-wise type I error rate of 0.01 by running 1000 permutations. QTL class analysis was performed by generating QTL classes with various allele combinations for the two most consistent QTLs (qAG6–2 and qAG10–1) identified in the study and comparing the mean of these classes to determine the interaction patterns and most advantageous allele combination. The analysis was limited to two of these QTLs due to lack of enough number of lines per class when all four QTLs were considered.

## Data Availability

The datasets used and/or analyzed during the current study are available from the corresponding author on request.

## Abbreviations

AG: Anaerobic germination
AMMI: Additive main effects and multiplicative interactions
ANOVA: One-way analysis of variance
BSA: Bulk segregant analysis
CIM: Composite interval mapping
DAS: Days after seeding
GXE: Genotype-by-environment interaction
PC: Principal component
PCR: Polymerase chain reaction
QTLs: Quantitative trait loci
SG: Selective genotyping
SNP: Single nucleotide polymorphism
SSR: Simple sequence repeat
WGS: Whole genome sequencing
